# Effect of TCDD exposure in adult female and male mice on the expression of miRNA in the ovaries and testes and associated reproductive functions

**DOI:** 10.3389/ftox.2023.1268293

**Published:** 2023-10-03

**Authors:** Alina Hall, Donald Mattison, Narendra Singh, Ioulia Chatzistamou, Jiajia Zhang, Mitzi Nagarkatti, Prakash Nagarkatti

**Affiliations:** ^1^ Department of Pathology, Microbiology and Immunology, School of Medicine, University of South Carolina, Columbia, SC, United States; ^2^ Arnold School of Public Health, University of South Carolina, Columbia, SC, United States

**Keywords:** TCDD, miRNA, reproductive toxicity, ovary, testes

## Abstract

2,3,7,8-Tetrachlorodibenzo-p-dioxin (TCDD) is an environmental contaminant found widely across the world. While animal and human studies have shown that exposure to TCDD may cause significant alterations in the reproductive tract, the effect of TCDD on the expression of miRNA in the reproductive organs has not been previously tested. In the current study, we exposed adult female or male mice to TCDD or vehicle and bred them to study the impact on reproduction. The data showed that while TCDD treatment of females caused no significant change in litter size, it did alter the survival of the pups. Also, TCDD exposure of either the male or female mice led to an increase in the gestational period. While TCDD did not alter the gross morphology of the ovaries and testes, it induced significant alterations in the miRNA expression. The ovaries showed the differential expression of 426 miRNAs, of which 315 miRNAs were upregulated and 111 miRNA that were downregulated after TCDD exposure when compared to the vehicle controls. In the testes, TCDD caused the differential expression of 433 miRNAs, with 247 miRNAs upregulated and 186 miRNAs downregulated. Pathway analysis showed that several of these dysregulated miRNAs targeted reproductive functions. The current study suggests that the reproductive toxicity of TCDD may result from alterations in the miRNA expression in the reproductive organs. Because miRNAs also represent one of the epigenetic pathways of gene expression, our studies suggest that the transgenerational toxicity of TCDD may also result from dysregulation in the miRNAs.

## Introduction

2,3,7,8-tetrachlorodibenzo-p-dioxin (TCDD), is an environmental pollutant that is considered to be the most persistent and strongest endocrine disruptor (Hites, 2011; Zhang et al., 2021). TCDD is also a historically controversial chemical contaminant that was produced during the high-temperature synthesis of organochloride herbicides, such as Agent Orange, which was heavily utilized during the Vietnam War (Kim et al., 2003; NASEM, 2018). Veterans exposed to TCDD contamination in Agent Orange during the war have exhibited an increased occurrence of various cancers (Chang et al., 2014; Chang et al., 2017), dementia (Martinez et al., 2021), skin disorders like porphyria and chloracne (Patterson et al., 2016), as well as endocrine and reproductive system disease (Gaspari et al., 2021).

TCDD toxicity is mediated through the aryl hydrocarbon receptor (AhR) which is translocated to the nucleus after ligand binding. Here, it forms a heterodimer with the aryl hydrocarbon receptor nuclear translocator (ARNT) protein which can then directly interact with genes containing Dioxin Response Elements (DREs) (Enan and Matsumara, 1996; Rothhammer and Quintana, 2019). These DREs alter gene expression resulting in changes in physiopathology including both immunosuppressive and inflammatory processes (Clark et al., 1981; Dominques-Acosta et al., 2018; Vasquez-Gomez et al., 2022). TCDD exposure has been observed to disrupt normal immune system development and functions such as increasing thymic atrophy (De Heer et al., 1994; Frazier et al., 1994; Kamath et al., 1997; Camacho et al., 2005), alterations to thymocyte development (Fine et al., 1990) and increased T-cell apoptosis (Singh et al., 2020). Additionally, hormone synthesis and circulation disruption has also been observed, following TCDD exposure, in the thyroid (Kohn, 2000), hypothalamus (Fetissov et al., 2004), pituitary and adrenal glands (Bestervelt et al., 1993).

In addition to modulating gene expression and hormone activity, direct effects on reproductive success have been observed after exposure to dioxin in murine models. Previous research from our laboratory has revealed TCDD induces a loss of mitochondrial membrane potential in epididymal spermatozoa, potentially reducing sperm quality and motility (Fisher et al., 2005) as well as reducing testes' weight and circulating testosterone levels (Choi et al., 2008; Zhang et al., 2021). Paternal exposure to TCDD has been shown to distort the sex ratio of the offspring, increasing the proportion of females per litter (Bircsak et al., 2020). Females exposed to TCDD had increased rates of endometriosis and ovarian cancers (Johnson et al., 1997; Bruner-Tran et al., 2017) as well as altered ovarian function (Tischkau et al., 2011), and reduced estradiol synthesis (Valdez et al., 2009). Epigenetic changes induced by TCDD are heritable and directly observable at the germ line (Patrizi and Siciliani de Cumis, 2018). By studying these alterations at the gonad level, one can better characterize the long-term and transgenerational risk of exposure.

Increased expression of genes containing DREs is not the only transcriptional disruption induced by TCDD. TCDD can also alter the expression of mRNA through dysregulation of miRNA (Singh et al., 2012; AlGhezi et al., 2019; Neamah et al., 2019) which are short, ∼20 bp long, non-coding RNAs that regulate gene translation by silencing target mRNA sequences (O’Brien et al., 2018). However, some miRNA express DREs in their 3′ UTR, thereby enabling TCDD to directly alter their expression through DRE-dependent pathways (Disner et al., 2021). miRNAs serve as critical epigenetic modulators, altering the protein levels of the target mRNAs without changing the gene sequences (Yao et al., 2019).

The effect of *in vivo* exposure to TCDD on miRNA expression in the reproductive organs and its impact on reproductive functions has not been previously studied. However, there are a few reports on the *in vitro* effects of TCDD on miRNA expression in cells such as Chinese Hamster Ovary (CHO) cells (Sadowska, et al., 2021) and human Sertoli cells (Ribeiro, et al., 2018), the former study showing no significant changes in miRNA expression while the latter showing significant alterations. The present study investigated the miRNA expression in the ovaries and testes of adult mice exposed to TCDD and the genes targeted by those miRNAs. Our data demonstrate that TCDD alters the expression of a large number of miRNAs and several of them target the gene expression involved in the regulation of reproductive functions.

## Materials and methods

### Mice

Male and Female C57BL/6J mice aged 5–6 weeks were purchased from Jackson Laboratories (Bar Harbor, ME, United States 04609) and housed at the AAALAC-accredited DLAR animal facility at the University of South Carolina School of Medicine (Columbia, SC, United States 20208) under specific pathogen-free conditions. Before and throughout the experiment, animals were maintained in temperature and humidity-controlled rooms on 12-h light/dark cycles and provided with access to food and normal rodent chow *ad libitum.* Female mice were housed together in cages of five without male contact before random pairing and/or treatment to allow for estrus cycles to synchronize. All experiments were performed in accordance with ethical standards approved by the University of South Carolina Institutional Animal Care and Use Committee.

### TCDD exposure and mating pairs

**FIGURE 1:**
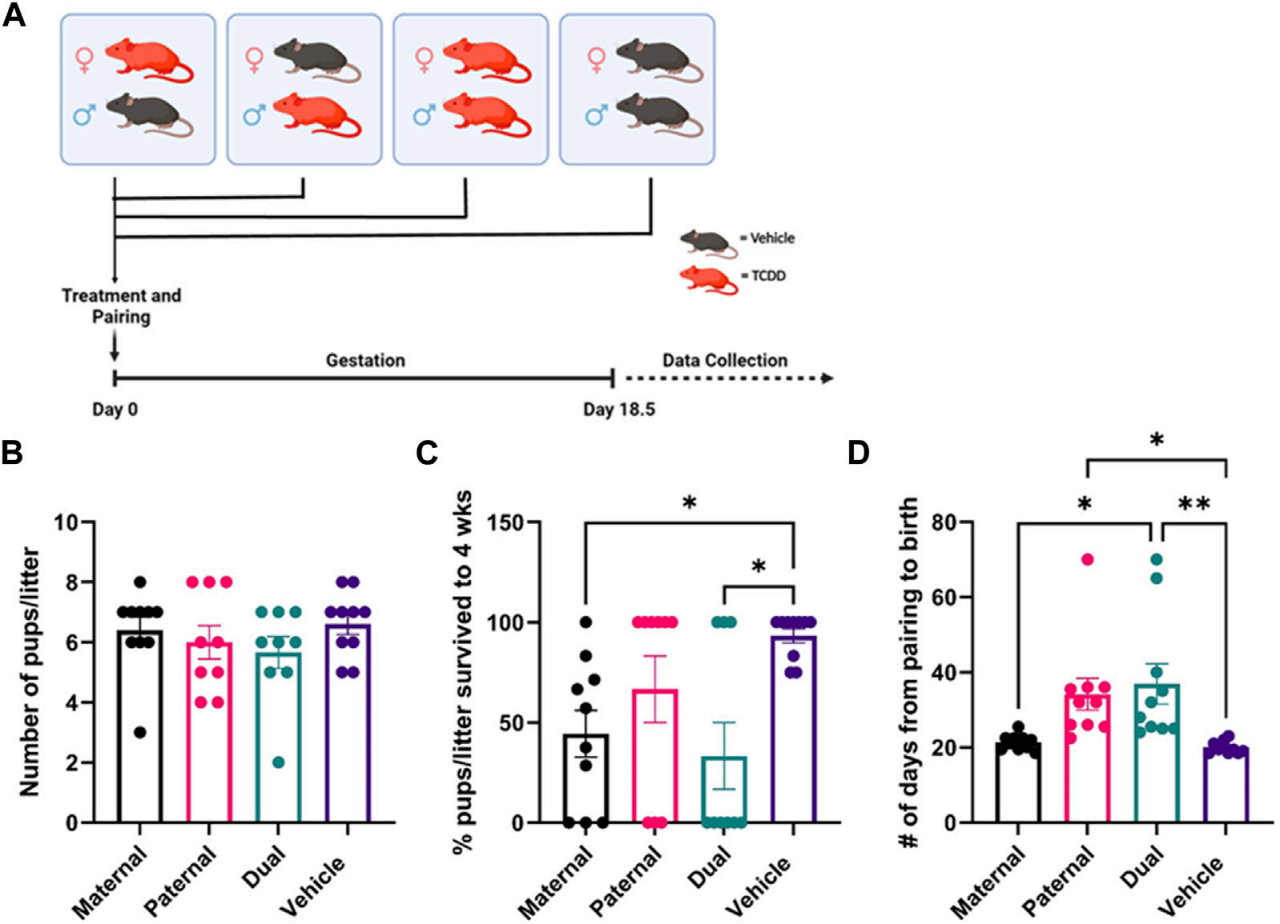
Effect of TCDD exposure on reproductive functions. There were four groups of mice: 1) Adult female mice treated with TCDD and mated with male mice treated with vehicle, 2) Adult female mice treated with vehicle and mated with male mice treated with TCDD, 3) Both male and female mice treated with TCDD, 4) Both male and female mice treated with vehicle. Such mice were then paired and following pregnancy, the pregnant mice were observed for the number of pups delivered, the percentage of pup survival per litter for up to 4 weeks, and the number of days taken from the sighting of vaginal plug to the birth of the pups. Reproductive success parameters by treatment group were measured as follows: **(B)** litter size at the time of birth, **(C)** post-natal pup survivorship to weaning age, **(D)** time from pairing to birth. Significance was determined using Student’s t-test with *n* = 10. The asterisk denotes as follows: * <0.05, **<0.01.

TCDD was generously provided by Dr. Steve Safe (Institute of Biosciences & Technology, Texas A&M Health Science Center, College Station, TX, United States) and was dissolved in DMSO to a working stock concentration of 100 μg/mL. The stock was combined with medical-grade corn oil for a total volume of 100 µL injected intraperitoneally (I.P). Mice were dosed at 10 μg/kg body weight similar to other studies which have used similar doses (Besteman, et al., 2007; Li, et al., 2022). Male and female mice were either treated with TCDD or vehicle, the latter consisting of an equal amount of DMSO in corn oil, then immediately paired with one male to one female per cage for initial reproductive success assessment. Treatment and pairing design are described in Figure 1A. For miRNA expression analysis, male and female mice were treated as previously described, kept with their original same-sex cage mates, and sacrificed 72-h post-exposure.

### Reproductive parameter assessment

After treatment and pairing, the female mice were examined for the vaginal plug, the sighting of which was considered as possible day 1 of pregnancy. Next, the mated pairs were left undisturbed until females were visibly pregnant at which time males were removed for parturition. Daily monitoring of females was conducted and measurements such as days since pairing, number of pups born in a litter, and number of neonates found deceased in the cage were recorded until weaning age was reached at 4 weeks.

### Histology

Mice were euthanized with an overdose of inhaled isoflurane and testes and ovaries were excised from the male and female mice, respectively, rinsed with PBS, and immersed in 4% paraformaldehyde (PFA). Paraffin embedding, cutting, and mounting, and H&E staining was performed on-site through the University of South Carolina School of Medicine Instrument Resource Facility (IRF). Imaging was conducted on a Keyence BZ-X800 Automated High-resolution Fluorescence Microscope (Keyence Corporation of America, Itasca, IL, United States 60143).

### Tissue processing and miRNA isolation

Testes and ovaries were excised and placed in blender bags containing RPMI medium containing FBS (10% v/v) and an antibiotic cocktail including penicillin and streptomycin. All tissues were kept on ice during processing. Tissues were homogenized using a Seward ™ Stomacher ™ Model 80 Biomaster Blender 110 V (Seward Inc, Bohemia, NY, United States) and filtered using 100 µm mesh filters. The resulting filtrate was centrifuged at 350 × g for 7 min and the supernatant was discarded. The remaining cell pellet was resuspended in 700 µL of QIAzol ™ Lysis reagent (QIAGEN, Germantown, MD, United States 20874). miRNA was isolated using a QIAGEN miRNEasy Micro Kit (QIAGEN, Germantown, MD, United States 20874) according to the manufacturer’s instructions.

### miRNA microarray

miRNA arrays were performed as described previously (Al-Ghezi et al., 2019). Purified miRNA samples isolated from testes and ovaries harvested from mice 72 h post-exposure to TCDD were labeled using the Affymetrix^®^ FlashTag™ Biotin HSR RNA Labeling Kit using the manufacturer’s specifications for 100 format miRNA 4.0 arrays. GeneChip™ Hybridization, Wash, and Stain kit was used in conjunction with the Affymetrix^®^ Hybridization Oven and Affymetrix^®^ Fluidics Station on the 450 protocol to prepare Affymetrix^®^ GeneChip^®^ miRNA 4.0 arrays. Prepared arrays were then analyzed using the GeneChip™ Scanner 3,000 7G (All products in this section are sourced from ThermoFisher Scientific, Waltham, MA, United States 02451). Microarray intensity was quantified, and differential expression was analyzed using Applied Biosystems Transcription Analysis Console (TAC) 4.0 software. Expression data were analyzed using Ingenuity Pathway Analysis (IPA) and miRNA targets were determined using the current target prediction database miRDB (Chen and Wang, 2020), and target prediction algorithm, MirTarget as well as web-based TargetScan. MicroRNA raw data have been deposited to Geo database (accession number is GSE241576 and can be accessed by https://www.ncbi.nlm.nih.gov/geo/info/linking.html) of National Institute of Health (NIH).

### Real-time quantitative PCR (RT-qPCR) to validate the expression of miRNAs and genes

miRNAs and target mRNAs of interest were validated using Real-Time PCR. To synthesize cDNA, iScript™ cDNA Synthesis Kit from Bio-RAD (BIO-RAD, Herculeus, VCA) was used. RT-qPCR using SYBR^®^ Green qPCR Supermix from Qiagen (Qiagen, Valencia, CA) was used following the protocol of the company. Real-qPCR was performed on a BIO-RAD CFX Connect RT-PCR Detection System (BIO-RAD, Hercules, CA). RT-qPCR was performed for 40 cycles and using the following conditions: initial activation step (5 min at 95°C), denaturing temperature (15 s at 94°C), annealing temperature (30 s at 55°C), and extension temperature and fluorescence data collection (30 s at 70°C). Normalized expression (NE) of miRs and genes were calculated using NE ¼ 2_DDCt, where Ct is the threshold cycle to detect fluorescence. The expression of miRNAs was normalized against internal control for miRNA and fold change of miRNAs expression was calculated against the internal control SNORD96A, and the treatment group (TCDD) was compared with the vehicle group. Similarly, the value of genes was normalized against the housekeeping gene (18 S) and fold-change of genes was calculated against 18 S and the TCDD group was compared with the vehicle group. To define significant differences in the expression of miRNAs and genes, ANOVA was performed using GraphPad version 6.0 (GraphPad Software, Inc., San Diego, CA, United States), and differences between the groups were considered significant when *p* < 0.05.

### Data analysis and statistics

We used groups of 10 mice injected with TCDD or the vehicle which were then allowed to mate for studies on reproduction. In other studies, we used groups of 5 mice for studies on the ovaries and testes. For miRNA microarray analysis, we pooled the ovaries or testes from 5 animals while the validation of miRNA using RT-qPCR was performed on individual mice with *n* = 5. The statistical significance was determined using a one-way ANOVA followed by Tukey’s *post hoc* test.

## Results

### Reproductive success is modulated by parental exposure to TCDD

Wild type C57BL/6J male and female mice were exposed to Vehicle or TCDD as described in Methods, paired, and allowed to mate as described in Figure 1A. There were 4 groups of mice: 1) Female mice exposed to TCDD mated with male mice treated with Vehicle (Maternal). 2) Male mice exposed to TCDD mated with female mice treated with Vehicle (Paternal). 3) Both male and female mice exposed to TCDD (Dual). 4) Both male and female mice exposed to Vehicle only (Vehicle).

The pregnant mice were observed for the number of pups delivered, the percentage of pup survival per litter up to 4 weeks of observation, and the number of days taken from pairing to the birth of the pups. The maternal, paternal, or dual exposure to TCDD did not significantly alter the litter size (# of pups/litter) (Figure 1B). However, the survival of the pups was significantly impacted by TCDD exposure. Specifically, maternal but not paternal exposure to TCDD caused a significant decrease in the survival of pups to weaning age when compared to the Vehicle group (Figure 1C). The non-surviving pups were born alive, but it was unclear if subsequent post-natal mortality was due to health complications or live cannibalism by the mother. Additionally, the gestation period was significantly delayed following maternal exposure to TCDD but not after paternal exposure when compared to the Vehicle controls (Figure 1D). Together, these data suggested that maternal but not paternal exposure to TCDD significantly alters the pregnancy and the survival of the pups.

### TCDD induces changes in miRNA expression in the ovaries

**FIGURE 2:**
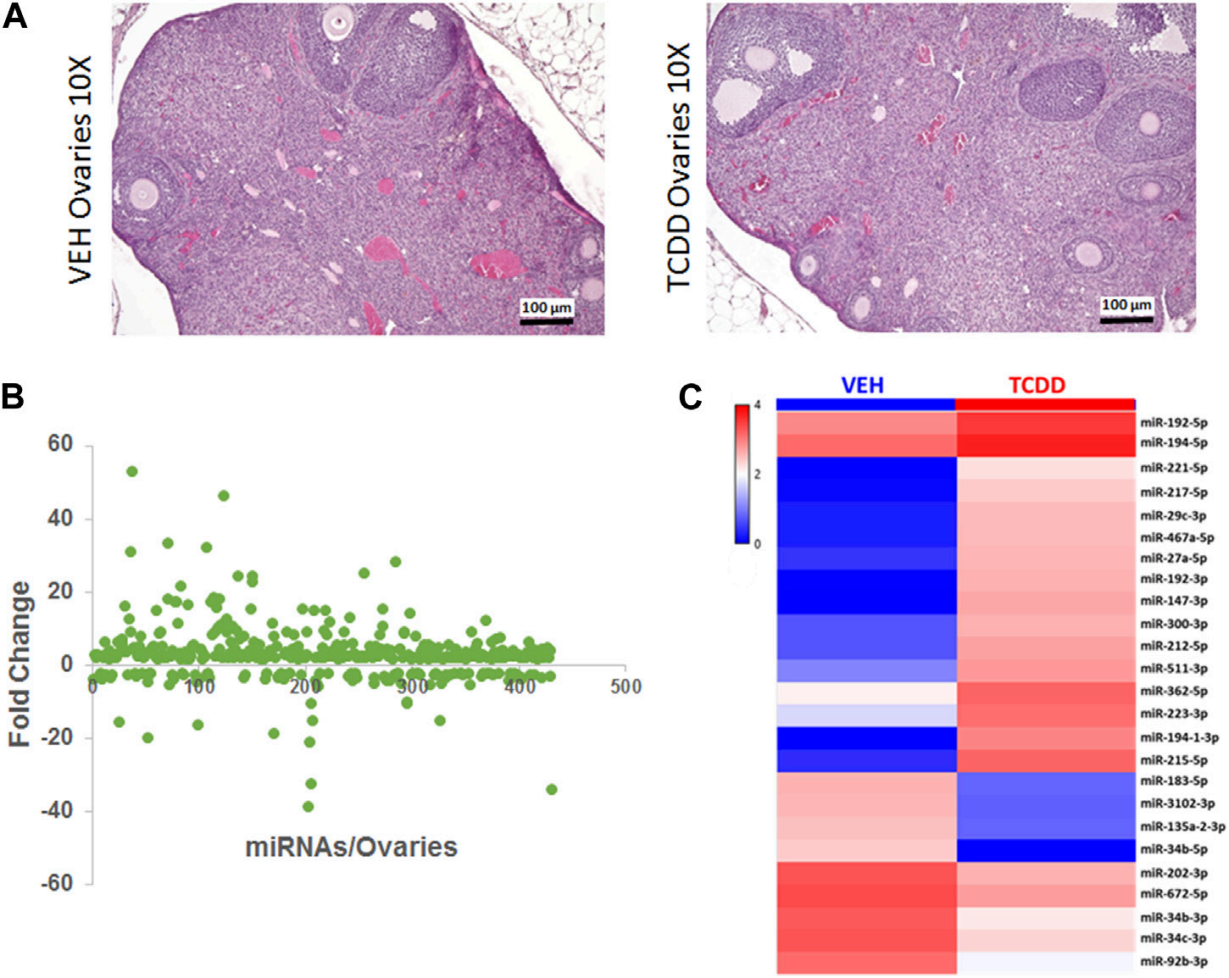
Effect of TCDD on histopathology and miRNA expression in the ovaries. Adult female mice were treated with TCDD or the Vehicle and 3 days later, the ovaries were harvested for analysis as described in Methods. **(A)** Mouse ovaries sections stained with H&E at ×10 magnification were collected from females 72 h after treatment with either Vehicle or TCDD. **(B)** GeneChip miRNA 4.0 microarray differential expression analysis of miRNA isolated from mouse ovaries. miRNAs with relative expression ≥2 were considered for downstream analysis. **(C)** Expression heat map of miRNA that were highly dysregulated in the mouse ovaries.

Gross and histologic examination of the ovaries of TCDD-treated mice showed no significant differences, and several ovarian follicles at various stages of development were observed in the cortex, that were similar in the TCDD and the Vehicle controls (Figure 2A). Next, we investigated if maternal exposure to TCDD would alter the expression of miRNA in the ovaries by using iGeneChip miRNA microarray. These studies revealed the differential expression of 426 miRNAs in the ovaries, of which 315 miRNAs were upregulated and 111 miRNAs were downregulated after TCDD exposure by ≥ 2-fold when compared to the Vehicle control (Figure 2B). The heatmap of some of the most dysregulated miRNAs has been depicted in Figure 2C, which shows marked differences between the two groups.

**FIGURE 3:**
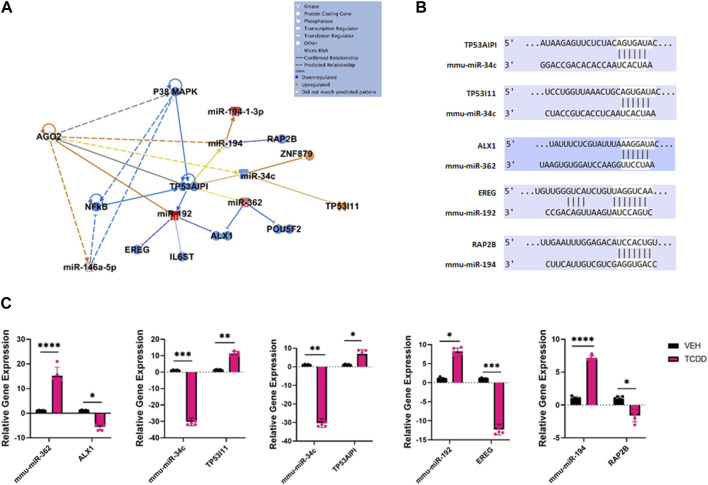
Characterization of TCDD-mediated alterations in miRNA expression in the ovaries and their predicted targets. **(A)** Ingenuity Pathway Analysis network of specific dysregulated miRNAs, downstream targets, and upstream regulators. **(B)** Predicted alignments of miRNA and downstream mRNA target sequences sourced from TargetScan. **(C)** RT-qPCR relative quantification of miRNAs of interest and potential mRNA targets in the ovaries. In Panel C, *n* = 5 and the Asterix denotes as follows: * <0.05, **<0.01, ***<0.001, ****<0.0001.

Ingenuity Pathway Analysis of some select highly dysregulated miRNA revealed how these miRNAs were predicted to target various upstream regulators and downstream mRNA targets involved with reproductive disease and normal fetal development (Figure 3A). The predicted miRNA/mRNA interactions were also confirmed through alignment using TargetScan (Figure 3B). For example, miR-34c was downregulated in the TCDD-treated group which targeted Tumor protein 53 apoptosis inducing protein 1 (TP53AIP1) and Tumor protein 53 inducible protein 11 (TP53I11), key regulatory molecules involved in the maintenance of homeostasis when the cells are in a state of stress and shown to be involved in preeclampsia (Ali et al., 2021).TCDD also decreased the levels of miR-192 which targeted epiregulin (EREG) known to stimulate oocytes (Romero and Smitz, 2009). TCDD upregulated miR-362 and miR-194 which targeted Alx Homebox Protein 1 (ALX1) and Ras family protein 2 B (RAP2B), respectively. Loss of ALX1 function is associated with birth defects (Iyyanar et al., 2022) while RAP2B is involved in cell proliferation and migration (Di et al., 2015).

To validate the miRNA array data and to confirm the effect of altered miRNA expression on the target genes, we performed RT-qPCR of miRNAs of interest and their potential targets (Figure 3C). The data showed that TCDD-exposed ovaries had decreased levels of miR-34c which correlated with increased expression of TP53AIPI and TP53I11 (Figure 3C). Additionally, TCDDD exposure led to increased expression of miR-362 and miR-194 which correlated with decreased expression of ALX1 and RAP2B (Figure 3C).

### TCDD induces changes in miRNA expression in the testes

**FIGURE 4:**
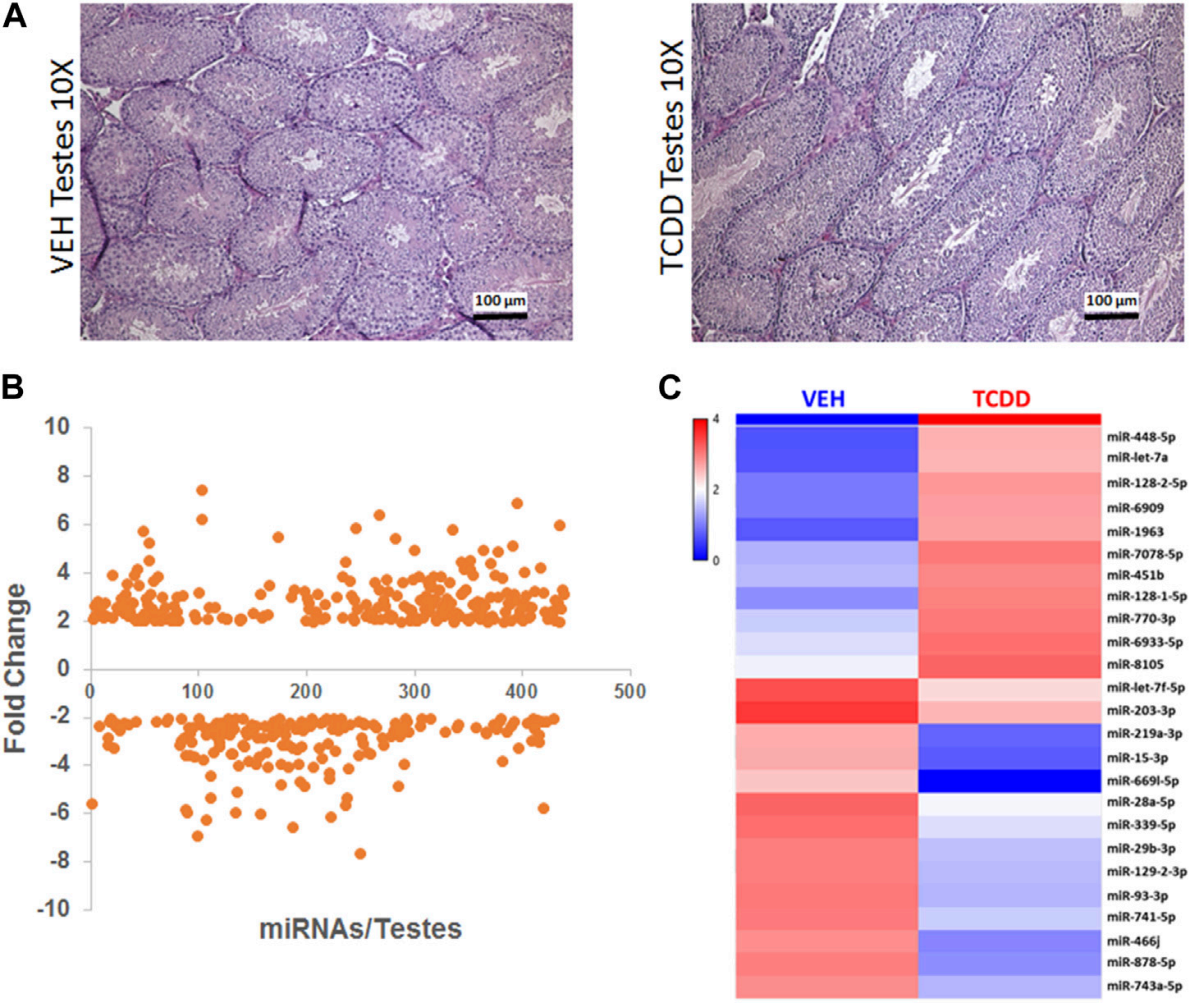
Effect of TCDD on histopathology and miRNA expression in the testes. Adult male mice were treated with TCDD or the Vehicle and 3 days later, the testes were harvested for analysis as described in Methods. **(A)** Mouse testes sections stained with H&E at ×10 magnification were collected from females 72 h after treatment with either Vehicle or TCDD. **(B)** GeneChip miRNA 4.0 microarray differential expression analysis of miRNA isolated from mouse testes. miRNAs with relative expression ≥2 were considered for analysis. **(C)** Expression heat map miRNA isolated from mouse testes that were highly dysregulated.

Next, we investigated the effect of TCDD exposure on miRNA expression in the testes. No significant differences in gross morphology of the testes from male mice treated with TCDD were observed when compared to the Vehicle controls, and histologically, the TCDD-treated groups showed normal spermatogenesis (Figure 4A).

Next, we investigated the effect of TCDD exposure on miRNA expression in the testes. Between males treated with Vehicle or TCDD, no significant differences in gross morphology were observed and normal spermatogenesis was noted in both (Figure 4A). GeneChip miRNA microarray of the testes revealed differential expression of 433 miRNAs, with 247 miRNAs upregulated and 186 miRNAs downregulated after exposure to TCDD (Figure 4B). Some of the highly dysregulated miRNAs following TCDD treatment have been depicted in the form of a heatmap in Figure 4C.

**FIGURE 5:**
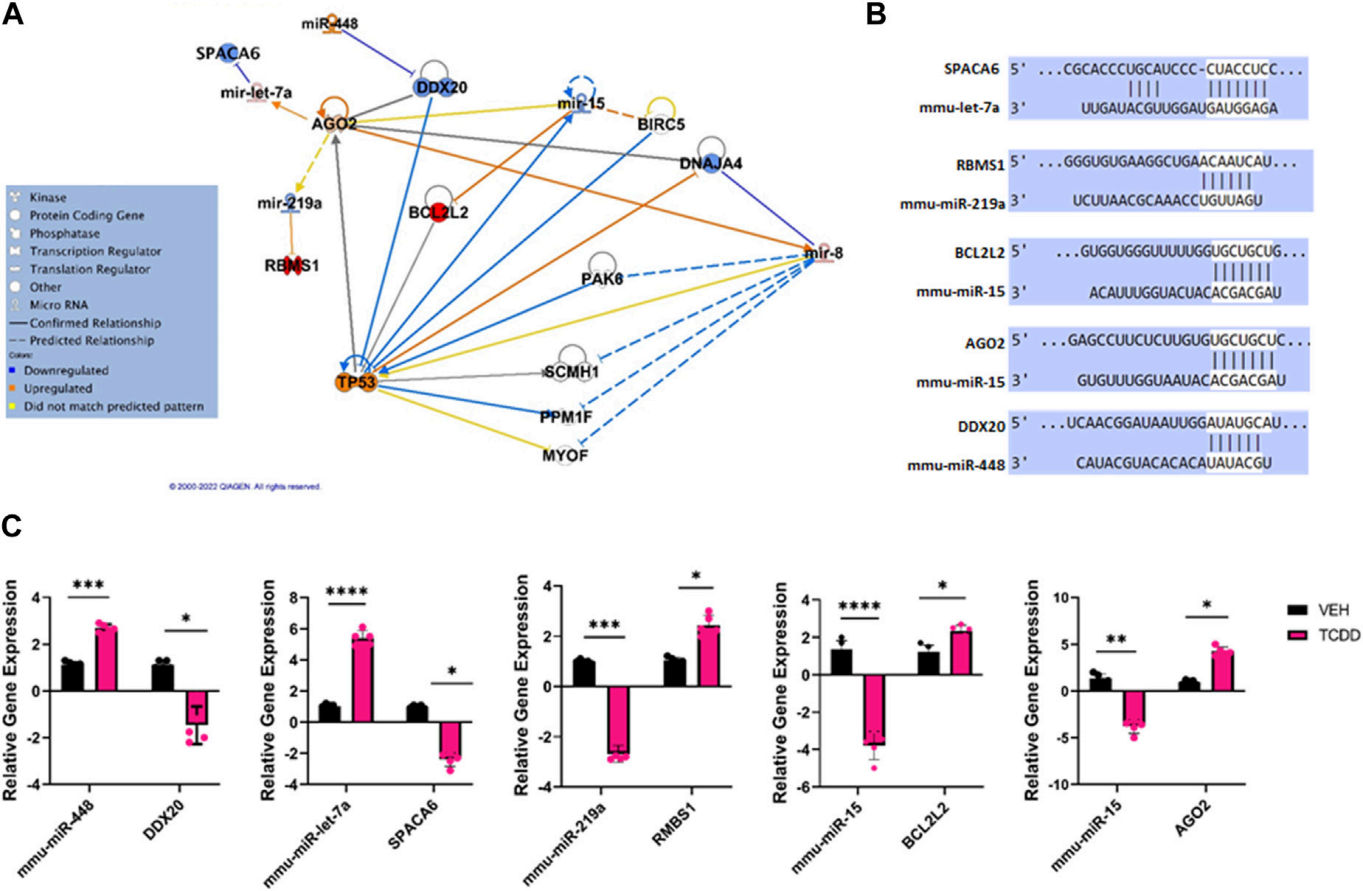
Characterization of TCDD-mediated alterations in miRNA expression and their predicted targets in the testes. **(A)** Ingenuity Pathway Analysis network of specific dysregulated miRNAs, downstream targets, and upstream regulators. **(B)** Predicted alignments of specific miRNA and downstream mRNA target sequences sourced from TargetScan. **(C)** RT-qPCR relative quantification of miRNAs of interest and potential mRNA targets in the testes. In Panel C, *n* = 5 and the Asterix denotes as follows: * <0.05, **<0.01, ***<0.001, ****<0.0001.

Ingenuity Pathway Analysis of some of the highly dysregulated miRNA showed targeting of genes involved in cell signaling pathways (Figure 5A). These dysregulated functional clusters revealed significant upregulation of certain miRs such as miR-448 and miR-let-7a as well as significant downregulation of miR-219a, and miR-15 and predicted various upstream regulators and downstream mRNA targets (Figure 5A). The predicted miRNA/mRNA interactions were aligned using TargetScan (Figure 5B). RT-qPCR of miRNAs of interest and potential mRNA targets revealed that upregulation of miRNA such as miR-448 and miR-let-7a was associated with decreased expression of specific targets such as DEAD-Box Helicase 20 (DDX20) and Sperm acrosome associated protein 6 (SPACA6), respectively. Moreover, decreased expression of miR-219a, and miR-15 was associated with increased expression of binding motif single-stranded interacting protein 1 (RBMS1), Bcl-like protein L2 (BCL2L2), and Argonaut RISC catalytic component 2 (AGO2).

### Comparison of miRNA alterations between testes and ovaries and pathway analysis of miRNA to predict a targeted effect on various disorders

**FIGURE 6:**
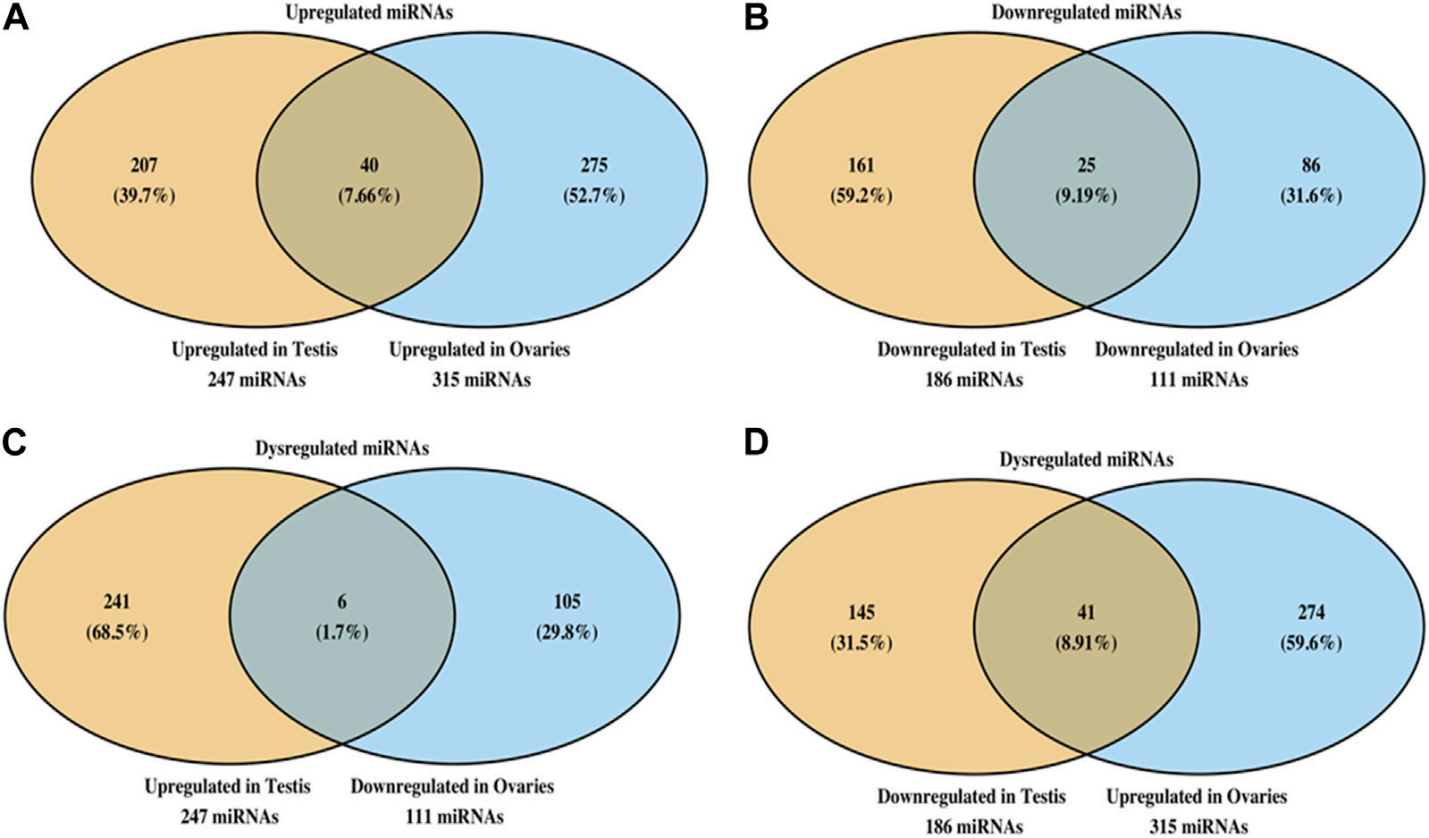
Analysis of miRNA showing similar or dissimilar alterations in the ovaries and testes. Venn diagram showing miRNA that are upregulated **(A)** or downregulated **(B)** in ovaries and testes. The data show the numbers of miRNA changes that are unique to each organ as well as shared by the two organs. Panel C shows sharing of miRNA that were upregulated in testes and downregulated in ovaries, and *vice versa* **(D)**.

When we compared the miRNA expression changes between the testes and the ovaries, we noted that the TCDD-exposed testes had 247 miRNA upregulated while the ovaries had 315 miRNA that were upregulated (Figure 6A). Also, of these upregulated miRNA, 7.66% shared similar upregulation among these two organs (Figure 6A). Also, the testes showed 186 miRNA to be downregulated following TCDD exposure while the ovaries showed 111 miRNA to be downregulated (Figure 6B). Additionally, these two organs showed 9.19% of such miRNA to be similarly downregulated (Figure 6B). Interestingly, only a small number (1.7%) of the dysregulated miRNA showed reciprocal alterations such as upregulation in the testes and downregulation in the ovaries (Figure 6C). Also, 8.91% of the dysregulated miRNA showed reciprocal alterations such as downregulation in the testes and upregulation in the ovaries (Figure 6D).

## Discussion

TCDD or dioxin is a highly prevalent environmental contaminant. It is created by natural events such as volcanic eruptions and forest fires, and waste incineration, paper bleaching using chlorine, and certain plastics manufacture (Zhang et al., 2015). It was also found as a contaminant in Agent Orange, an herbicide used extensively during the Vietnam War which led to significant exposure of the Vietnam War Veterans as well as Vietnam civilians (Scialli et al., 2015). A chemical factory explosion near Seveso, Italy exposed the residents to high levels of TCDD (Eskenazi et al., 2018). TCDD is considered to be a human carcinogen and a potent endocrine disruptor (Bruner-Tran et al., 2017). Because it is highly lipophilic, following exposure, it can stay for a very long period in the body thereby causing chronic toxicity (Huff et al., 1991). The half-life of TCDD in humans is estimated to be around 7–9 years (Pirkle et al., 1989). Reports on human exposures and experimental studies have revealed that TCDD exposure is associated with chloracne of the skin, organ cancers, hepatotoxicity, gonadal and immune changes, pulmonary and other diseases such as diabetes, skewing of the sex ratio, and infertility (Kociba and Schwetz, 1982; Birnbaum, 1994; Kamath et al., 1998; Kamath et al., 1999; Singh et al., 2007).

The ability of TCDD to act as an endocrine disruptor suggests that it can have a significant impact on reproductive health. In fact, animal studies have revealed that exposure to TCDD in adult female mice can lead to transgenerational disorders involving reproductive functions that have been linked to endometriosis and other defects in women (Bruner-Tran et al., 2017). In certain species, exposure to TCDD is known to cause decreased fecundity and reduced ovulatory rate. Similarly, in humans, TCDD exposure was associated with decreased fertility in Seveso mothers and their daughters exposed *in utero* (Eskenazi et al., 2021).

The current study revealed that exposure of adult female mice to TCDD was significantly linked to poor survivorship of neonates but had no effect on the litter size at birth. Also, TCDD exposure led to an increase in the gestational period. The decreased survival of the pups was seen only when adult female but not adult male mice were exposed to TCDD. However, the increase in the gestational period was seen when either both male or female mice were exposed to TCDD. This suggests that maternal TCDD exposure may interfere with normal pup development or induces postpartum stress which can trigger cannibalism. The precise cause of postnatal mortality in the mouse is unclear and it is suggested that causes other than infanticide play a critical role (Weber et al., 2013).

miRNAs constitute noncoding RNAs that have been shown to play a critical role in the posttranscriptional regulation of gene expression. In the reproductive system, the miRNAs can regulate oocyte maturation, folliculogenesis, corpus luteum function, implantation, and early embryonic development (Eisenberg et al., 2015). It has been shown that deletion of Dicer, the enzyme that cleaves the pre-miRNA to the mature form, leads to post-implantation embryonic lethality in many animal models, thereby suggesting that miRNA plays a critical role in reproduction and development (Meng et al., 2013).

miRNA is expressed in the ovaries and is known to regulate the formation of primordial follicles, oocyte-cumulus cell interactions, granulosal cell function, and luteinization (Maalouf et al., 2016). In the current study, TCDD exposure in female mice upregulated the expression of mmu-miR-362 in the ovaries and decreased the expression of its target, ALX1, a homeobox protein-coding gene necessary for the normal development of the head and face, especially the eyes, nose, and mouth (Iyyanar et al., 2022). Poor development of these critical features may compromise neonate health, thereby prompting the mothers to cannibalize the litter. Additionally, downregulation of miR-34c suppressed the normal post-transcriptional silencing of two genes TP53I11 and TP53AIPI, which play an important role in mediating p53-dependent apoptosis. P53 is a tumor protein that acts as a tumor suppressor and is negatively regulated by proteins encoded by both TP53I11 and TP53AIPI. Both genes were upregulated in the ovaries, possibly as a response to the teratogenic and oncogenic nature of TCDD. TP53I11 and TP53AIPI have also been shown to regulate homeostasis when the cells are in a state of stress and shown to be involved in preeclampsia (Ali et al., 2021). Additionally, miR-192 was upregulated in TCDD-exposed ovaries while downstream target EREG was significantly downregulated. EREG is involved with various biological processes but may also be associated with promoting cancers in various tissues (Maalouf et al., 2016). In the reproductive system, EREG is known to stimulate oocytes (Romero and Smitz, 2009). Likewise, miR-194 was significantly upregulated in TCDD-treated females while downstream target RAP2B was suppressed. RAP2B is a protein-encoding gene in the RAS oncogene family and is involved with the proliferation and migration of various cancer types (Di et al., 2016; Di et al., 2017; Zhang et al., 2017; Li et al., 2018; Miao et al., 2019).

Recent studies have shown that miRNAs play a critical role in spermatogenesis, especially during mitotic, meiotic, and post-meiotic stages of spermatogenesis (Wang and Xu, 2015). In the current study, we found that while exposure of male mice to TCDD did not alter the litter size or the survival of the pups, there was an increase in the time from pairing to parturition. Upregulation of two select miRNAs; miR-448 and mir-let-7a and the silencing of their downstream targets, DDX20 and SPACA6, respectively, may be involved in altering the testicular functions. DDX20 is a DEAD box protein-encoding gene that is involved in several cellular activities and based on distribution patterns, may be involved in spermatogenesis and embryogenesis (Lee et al., 2005). SPACA6 is a sperm acrosome membrane-associated protein directly involved in the fusion of sperm to egg plasma membrane (Barbaux et al., 2020).

Additionally, downregulation of two miRNAs; miR-219a and miR-15 in the testes by TCDD correlated with significant upregulation in their respective downstream targets RBMS1, and BCL2L2, as well as AGOT2 involved with cellular proliferation and apoptosis. BCL2L2 has additionally been shown to be critical in adult spermatogenesis and dysregulation of BCL-2 family proteins can induce germ cell apoptosis (Vergara et al., 2011). Lastly, while miR-15 targeted AGO2, it was suggested to act as an upstream regulator of miR-15, and AGO2 was also dysregulated in the ovaries treated with TCDD. This suggested an inverse relationship between AGOT and miR-15. Also, the pathway analysis of all dysregulated miRNA in the ovaries and testes revealed that these miRNA alterations affected to a greater extent, reproductive system diseases, organismal injury and abnormalities, and cancer, consistent with previous studies showing TCDD-mediated systemic toxicities.

AhR activation by TCDD leads to two distinct pathways of the regulation of gene expression: the canonical pathway and the non-canonical pathway (Wright, et al., 2017). In the canonical pathway, the AhR upon ligation, dimerizes with the AhR nuclear translocator (ARNT) and subsequently interacts with DREs leading to the induction of the genes that express DREs. In the non-canonical pathway, the AhR forms a complex with other molecules allowing it to bind to genes lacking DRE sequences leading to either induction or repression of gene expression (Viluksela and Pohjanvirta, 2019; Alhamad, et al., 2022). While the precise mechanisms through which TCDD regulates miRNA in this study is unclear, we and others have shown previously that some miRNAs express dioxin response elements (DRE) in their 3’ UTR and the induction of such miRNAs occurs through DRE-dependent pathway (Neamah, et al., 2019; Singh, et al., 2020). In the current study, we investigated the miRNAs for the expression of DREs using *in silico* analysis and found interestingly that all the miRNA that were altered by TCDD (Figures 3, 5) such as miR-362, -34c, -192 -194, -448, -let-7a, and -15, expressed DREs. Thus, TCDD may use canonical pathway to regulate miRNA expression in the reproductive organs, although the use of non-canonical pathways cannot be ruled out.

## Conclusion

The current study demonstrates for the first time that exposure of adult male and female mice to TCDD results in significant alterations in a large number of miRNAs in the ovaries and the testes. These findings suggest that environmental contaminants such as TCDD may alter reproductive functions through alterations in the expression of miRNAs. Future studies will focus on whether such miRNA changes also occur in the germ cells which would explain how TCDD exerts transgenerational effects on the reproductive system by regulating epigenetic pathways involving miRNA.

## Data Availability

MicroRNA raw data have been deposited to Geo database (accession number is GSE241576 and can be accessed by https://www.ncbi.nlm.nih.gov/geo/info/linking.html) of National Institute of Health (NIH).

## References

[B2] Al-Ghezi Z. Z. Singh N. Mehrpouya-Bahrami P. Busbee P. B. Nagarkatti M. Nagarkatti P. S. (2019). AhR activation by TCDD (2,3,7,8-tetrachlorodibenzo-p-dioxin) attenuates pertussis toxin-induced inflammatory responses by differential regulation of tregs and Th17 cells through specific targeting by microRNA. Front. Microbiol. 10, 2349. 10.3389/fmicb.2019.02349 31681214 PMC6813193

[B3] Alhamad D. W. Bensreti H. Dor J. Hill W. D. HamrickMcGee-Lawrence M. W. M. E. (2022). Aryl hydrocarbon receptor (AhR)-mediated signaling as a critical regulator of skeletal cell biology. J. Mol. Endocrinol. 69, R109–R124. 10.1530/JME-22-0076 35900841 PMC9448512

[B4] Ali Z. Zafar U. Zaki S. Ahmad S. Khaliq S. Lone K. P. (2021). Expression levels of MiRNA-16, SURVIVIN and TP53 in preeclamptic and normotensive women. J. Pak Med. Assoc. 71 (9), 2208–2213. 10.47391/JPMA.1171 34580516

[B5] Barbaux S. Ialy-Radio C. Chalbi M. Dybal E. Homps-Legrand M. Do Cruzeiro M. (2020). Sperm SPACA6 protein is required for mammalian Sperm-Egg Adhesion/Fusion. Sci. Rep. 10 (1), 5335. 10.1038/s41598-020-62091-y 32210282 PMC7093486

[B6] Besteman E. G. Zimmerman K. L. Huckle W. R. Prater M. R. Gogal R. M. Holladay S. D. (2007). 2,3,7,8-tetrachlorodibenzo-p-dioxin (TCDD) or diethylstilbestrol (DES) cause similar hematopoietic hypocellularity and hepatocellular changes in murine fetal liver, but differentially affect gene expression. Toxicol. Pathol. 35 (6), 788–794. 10.1080/01926230701584155 17943652

[B7] Bestervelt L. L. Pitt J. A. Nolan C. J. Piper W. N. (1993). TCDD alters pituitary-adrenal function. II: evidence for decreased bioactivity of ACTH. Neurotoxicol Teratol. 15 (6), 371–376. 10.1016/0892-0362(93)90053-q 8302237

[B8] Bircsak K. M. Copes L. T. King S. Prantner A. M. Hwang W. T. Gerton G. L. (2020). The aryl hydrocarbon receptor mediates sex ratio distortion in the embryos sired by TCDD-exposed male mice. Reprod. Toxicol. 94, 75–83. Epub 2020 Apr 23. 10.1016/j.reprotox.2020.04.072 32335222 PMC7303002

[B9] Birnbaum L. S. (1994). The mechanism of dioxin toxicity: relationship to risk assessment. Environ. Health Perspect. 102, 157–167. 10.1289/ehp.94102s9157 PMC1566802 7698077

[B10] Bruner-Tran K. L. Gnecco J. Ding T. Glore D. R. Pensabene V. Osteen K. G. (2017). Exposure to the environmental endocrine disruptor TCDD and human reproductive dysfunction: translating lessons from murine models. Reprod. Toxicol. 68, 59–71. 10.1016/j.reprotox.2016.07.007 27423904 PMC5237424

[B11] Camacho I. A. Singh N. Hegde V. L. Nagarkatti M. Nagarkatti P. S. (2005). Treatment of mice with 2,3,7,8-tetrachlorodibenzo-p-dioxin leads to aryl hydrocarbon receptor-dependent nuclear translocation of NF-kappaB and expression of Fas ligand in thymic stromal cells and consequent apoptosis in T cells. J. Immunol. 175 (1), 90–103. 10.4049/jimmunol.175.1.90 15972635

[B12] Chang C. Benson M. Fam M. M. (2017). A review of Agent Orange and its associated oncologic risk of genitourinary cancers. Urol. Oncol. 35 (11), 633–639. 10.1016/j.urolonc.2017.08.029 28947305

[B13] Chang E. T. Boffetta P. Adami H. O. Cole P. Mandel J. S. (2014). A critical review of the epidemiology of Agent Orange/TCDD and prostate cancer. Eur. J. Epidemiol. 29 (10), 667–723. 10.1007/s10654-014-9931-2 25064616 PMC4197347

[B75] Chen Y. Wang X. (2020). miRDB: An online database for prediction of functional microRNA targets. Nucleic Acids Res. 48 (D1), D127–D131. 10.1093/nar/gkz757 31504780 PMC6943051

[B15] Choi J. S. Kim I. W. Hwang S. Y. Shin B. J. Kim S. K. (2008). Effect of 2,3,7,8-tetrachlorodibenzo-p-dioxin on testicular spermatogenesis-related panels and serum sex hormone levels in rats. BJU Int. 101 (2), 250–255. 10.1111/j.1464-410X.2007.07202.x 17868417

[B16] Clark D. A. Gauldie J. Szewczuk M. R. Sweeney G. (1981). Enhanced suppressor cell activity as a mechanism of immunosuppression by 2,3,7,8-tetrachlorodibenzo-p-dioxin. Proc. Soc. Exp. Biol. Med. 168 (2), 290–299. 10.3181/00379727-168-41275 6216485

[B17] De Heer C. Verlaan A. P. Penninks A. H. Vos J. G. Schuurman H. J. Van Loveren H. (1994). Time course of 2,3,7,8-tetrachlorodibenzo-p-dioxin (TCDD)-induced thymic atrophy in the Wistar rat. Toxicol. Appl. Pharmacol. 128 (1), 97–104. 10.1006/taap.1994.1185 8079361

[B18] Di J. Cao H. Tang J. Lu Z. Gao K. Zhu Z. (2016). Rap2B promotes cell proliferation, migration and invasion in prostate cancer. Med. Oncol. 33 (6), 58. 10.1007/s12032-016-0771-7 27154636

[B19] Di J. Gao K. Qu D. Yang J. Zheng J. (2017). Rap2B promotes angiogenesis via PI3K/AKT/VEGF signaling pathway in human renal cell carcinoma. Tumour Biol. 39 (7), 1010428317701653. 10.1177/1010428317701653 28691643

[B20] Di J. Huang H. Qu D. Tang J. Cao W. Lu Z. (2015). Rap2B promotes proliferation, migration, and invasion of human breast cancer through calcium-related ERK1/2 signaling pathway. Sci. Rep. 5, 12363. 10.1038/srep12363 26201295 PMC4512009

[B21] Disner G. R. Lopes-Ferreira M. Lima C. (2021). Where the aryl hydrocarbon receptor meets the microRNAs: literature review of the last 10 years. Front. Mol. Biosci. 8, 725044. 10.3389/fmolb.2021.725044 34746229 PMC8566438

[B22] Domínguez-Acosta O. Vega L. Estrada-Muñiz E. Rodríguez M. S. Gonzalez F. J. Elizondo G. (2018). Activation of aryl hydrocarbon receptor regulates the LPS/IFNγ-induced inflammatory response by inducing ubiquitin-proteosomal and lysosomal degradation of RelA/p65. Biochem. Pharmacol. 155, 141–149. Epub 2018 Jun 21. 10.1016/j.bcp.2018.06.016 29935959 PMC6594173

[B23] Eisenberg I. Kotaja N. Goldman-Wohl D. Imbar T. (2015). microRNA in human reproduction. Adv. Exp. Med. Biol. 888, 353–387. 10.1007/978-3-319-22671-2_18 26663192

[B24] Enan E. Matsumura F. (1996). Identification of c-Src as the integral component of the cytosolic Ah receptor complex, transducing the signal of 2,3,7,8-tetrachlorodibenzo-p-dioxin (TCDD) through the protein phosphorylation pathway. Biochem. Pharmacol. 52 (10), 1599–1612. 10.1016/s0006-2952(96)00566-7 8937476

[B25] Eskenazi B. Ames J. Rauch S. Signorini S. Brambilla P. Mocarelli P. (2021). Dioxin exposure associated with fecundability and infertility in mothers and daughters of Seveso, Italy. Italy. Hum. Reprod. 36 (3), 794–807. 10.1093/humrep/deaa324 33367671 PMC7891815

[B26] Eskenazi B. Warner M. Brambilla P. Signorini S. Ames J. Mocarelli P. (2018). The Seveso accident: A look at 40 years of health research and beyond. Environ. Int. 121 (1), 71–84. 10.1016/j.envint.2018.08.051 30179766 PMC6221983

[B27] Fetissov S. O. Huang P. Zhang Q. Mimura J. Fujii-Kuriyama Y. Rannug A. (2004). Expression of hypothalamic neuropeptides after acute TCDD treatment and distribution of Ah receptor repressor. Regul. Pept. 119 (1-2), 113–124. 10.1016/j.regpep.2004.01.009 15093705

[B28] Fine J. S. Silverstone A. E. Gasiewicz T. A. (1990). Impairment of prothymocyte activity by 2,3,7,8-tetrachlorodibenzo-p-dioxin. J. Immunol. 144 (4), 1169–1176. 10.4049/jimmunol.144.4.1169 2303704

[B29] Fisher M. T. Nagarkatti M. Nagarkatti P. S. (2005). Aryl hydrocarbon receptor-dependent induction of loss of mitochondrial membrane potential in epididydimal spermatozoa by 2,3,7,8-tetrachlorodibenzo-p-dioxin (TCDD). Toxicol. Lett. 157 (2), 99–107. 10.1016/j.toxlet.2005.01.008 15836997

[B30] Frazier D. E. Jr Silverstone A. E. Gasiewicz T. A. (1994). 2,3,7,8-Tetrachlorodibenzo-p-dioxin-induced thymic atrophy and lymphocyte stem cell alterations by mechanisms independent of the estrogen receptor. Biochem. Pharmacol. 47 (11), 2039–2048. 10.1016/0006-2952(94)90079-5 8010988

[B31] Gaspari L. Paris F. Kalfa N. Soyer-Gobillard M. O. Sultan C. Hamamah S. (2021). Experimental evidence of 2,3,7,8-Tetrachlordibenzo-p-Dioxin (TCDD) transgenerational effects on reproductive health. Int. J. Mol. Sci. 22 (16), 9091. 10.3390/ijms22169091 34445797 PMC8396488

[B32] Hites R. A. (2011). Dioxins: an overview and history. Environ. Sci. Technol. 45 (1), 16–20. 10.1021/es1013664 20815379

[B33] Huff J. E. Salmon A. G. Hooper N. K. Zeise L. (1991). Long-term carcinogenesis studies on 2,3,7,8-tetrachlorodibenzo-p-dioxin and hexachlorodibenzo-p-dioxins. Cell Biol. Toxicol. 7 (1), 67–94. 10.1007/BF00121331 2054688

[B34] Iyyanar P. P. R. Wu Z. Lan Y. Hu Y. C. Jiang R. (2022). *Alx1* deficient mice recapitulate craniofacial phenotype and reveal developmental basis of *ALX1*-related frontonasal dysplasia. Front. Cell Dev. Biol. 10, 777887. 10.3389/fcell.2022.777887 35127681 PMC8815032

[B35] Johnson K. L. Cummings A. M. Birnbaum L. S. (1997). Promotion of endometriosis in mice by polychlorinated dibenzo-p-dioxins, dibenzofurans, and biphenyls. Environ. Health Perspect. 105 (7), 750–755. 10.1289/ehp.97105750 9294722 PMC1470109

[B36] Kamath A. B. Camacho I. Nagarkatti P. S. Nagarkatti M. (1999). Role of fas-fas ligand interactions in 2,3,7,8-tetrachlorodibenzo- p-dioxin (TCDD)-induced immunotoxicity: increased resistance of thymocytes from fas-deficient (lpr) and fas ligand-defective (gld) mice to TCDD-induced toxicity. Toxicol. Appl. Pharmacol. 160 (2), 141–155. 10.1006/taap.1999.8753 10527913

[B37] Kamath A. B. Nagarkatti P. S. Nagarkatti M. (1998). Characterization of phenotypic alterations induced by 2,3,7,8-tetrachlorodibenzo-p-dioxin on thymocytes *in vivo* and its effect on apoptosis. Toxicol. Appl. Pharmacol. 150 (1), 117–124. 10.1006/taap.1998.8390 9630460

[B38] Kamath A. B. Xu H. Nagarkatti P. S. Nagarkatti M. (1997). Evidence for the induction of apoptosis in thymocytes by 2,3,7,8-tetrachlorodibenzo-p-dioxin *in vivo* . Toxicol. Appl. Pharmacol. 142 (2), 367–377. 10.1006/taap.1996.8049 9070360

[B39] Kim H. A. Kim E. M. Park Y. C. Yu J. Y. Hong S. K. Jeon S. H. (2003). Immunotoxicological effects of agent Orange exposure to the Vietnam war Korean veterans. Ind. Health 41 (3), 158–166. 10.2486/indhealth.41.158 12916745

[B40] Kociba R. J. Schwetz B. A. (1982). Toxicity of 2, 3, 7, 8-tetrachlorodibenzo-p-dioxin (TCDD). Drug Metab. Rev. 13 (3), 387–406. 10.3109/03602538209029986 6213397

[B41] Kohn M. C. (2000). Effects of TCDD on thyroid hormone homeostasis in the rat. Drug Chem. Toxicol. 23 (1), 259–277. 10.1081/dct-100100114 10711401

[B42] Lee M. B. Lebedeva L. A. Suzawa M. Wadekar S. A. Desclozeaux M. Ingraham H. A. (2005). The DEAD-box protein DP103 (Ddx20 or Gemin-3) represses orphan nuclear receptor activity via SUMO modification. Mol. Cell Biol. 25 (5), 1879–1890. 10.1128/MCB.25.5.1879-1890.2005 15713642 PMC549377

[B43] Li J. Li Y. Sha R. Zheng L. Xu L. Xie H. Q. (2022). Effects of perinatal TCDD exposure on colonic microbiota and metabolism in offspring and mother mice. Sci. Total Environ. 832, 154762. 10.1016/j.scitotenv.2022.154762 35364153

[B44] Li Y. Li S. Huang L. (2018). Knockdown of Rap2B, a Ras superfamily protein, inhibits proliferation, migration, and invasion in cervical cancer cells via regulating the ERK1/2 signaling pathway. Oncol. Res. 26 (1), 123–130. 10.3727/096504017X14912172235777 28390112 PMC7844554

[B45] Maalouf S. W. Liu W. S. Pate J. L. (2016). MicroRNA in ovarian function. Cell Tissue Res. 363 (1), 7–18. 10.1007/s00441-015-2307-4 26558383

[B46] Martinez S. Yaffe K. Li Y. Byers A. L. Peltz C. B. Barnes D. E. (2021). Agent Orange exposure and dementia diagnosis in US veterans of the Vietnam era. JAMA Neurol. 78 (4), 473–477. 10.1001/jamaneurol.2020.5011 33492338 PMC7835948

[B47] Meng D. M. Wang L. Xu J. R. Yan S. L. Zhou L. Mi Q. S. (2013). Fabp4-Cre-mediated deletion of the miRNA-processing enzyme Dicer causes mouse embryonic lethality. Acta Diabetol. 50 (5), 823–824. 10.1007/s00592-011-0335-4 21984007

[B48] Miao F. Cui C. Zuo D. Zhang H. Mei P. Chen H. (2019). Rap2B promotes cell adhesion, proliferation, migration and invasion of human glioma. J. Neurooncol 143 (2), 221–229. 10.1007/s11060-019-03163-6 30997639

[B49] National Academies of Sciences (2018). Engineering, and medicine; health and medicine division; board on population health and public health practice; committee to review the health effects in Vietnam Veterans of exposure to herbicides (eleventh biennial update). Veterans and agent Orange: Update 11. Washington (DC): National Academies Press.

[B50] Neamah W. H. Singh N. P. Alghetaa H. Abdulla O. A. Chatterjee S. Busbee P. B. (2019). AhR activation leads to massive mobilization of myeloid-derived suppressor cells with immunosuppressive activity through regulation of CXCR2 and MicroRNA miR-150-5p and miR-543-3p that target anti-inflammatory genes. J. Immunol. 203 (7), 1830–1844. 10.4049/jimmunol.1900291 31492743 PMC6755129

[B51] O'Brien J. Hayder H. Zayed Y. Peng C. (2018). Overview of MicroRNA biogenesis, mechanisms of actions, and circulation. Front. Endocrinol. (Lausanne) 9, 402. 10.3389/fendo.2018.00402 30123182 PMC6085463

[B52] Patrizi B. Siciliani de Cumis M. (2018). TCDD toxicity mediated by epigenetic mechanisms. Int. J. Mol. Sci. 19 (12), 4101. 10.3390/ijms19124101 30567322 PMC6320947

[B53] Patterson A. T. Kaffenberger B. H. Keller R. A. Elston D. M. (2016). Skin diseases associated with Agent Orange and other organochlorine exposures. J. Am. Acad. Dermatol 74 (1), 143–170. 10.1016/j.jaad.2015.05.006 26210237

[B54] Pirkle J. L. Wolfe W. H. Patterson D. G. Needham L. L. Michalek J. E. Miner J. C. (1989). Estimates of the half-life of 2,3,7,8-tetrachlorodibenzo-p-dioxin in Vietnam veterans of operation ranch hand. J. Toxicol. Environ. Health 27 (2), 165–171. 10.1080/15287398909531288 2733058

[B55] Ribeiro M. A. Estill M. S. Fernandez G. J. Moraes L. N. Krawetz S. A. Scarano W. R. (2018). Integrative transcriptome and microRNome analysis identifies dysregulated pathways in human Sertoli cells exposed to TCDD. Toxicology 409, 112–118. 10.1016/j.tox.2018.08.001 30096437

[B56] Romero S. Smitz J. (2009). Epiregulin can effectively mature isolated cumulus-oocyte complexes, but fails as a substitute for the hCG/epidermal growth factor stimulus on cultured follicles. Reproduction 137 (6), 997–1005. 10.1530/REP-08-0523 19321658

[B57] Rothhammer V. Quintana F. J. (2019). The aryl hydrocarbon receptor: an environmental sensor integrating immune responses in health and disease. Nat. Rev. Immunol. 19 (3), 184–197. 10.1038/s41577-019-0125-8 30718831

[B58] Sadowska A. Nynca A. Ruszkowska M. Paukszto L. Myszczynski K. Swigonska S. (2021). Transcriptional profiling of Chinese hamster ovary (CHO) cells exposed to 2,3,7,8-tetrachlorodibenzo-p-dioxin (TCDD). Reprod. Toxicol. 104, 143–154. 10.1016/j.reprotox.2021.07.012 34363982

[B59] Scialli A. R. Watkins D. K. Ginevan M. E. (2015). Agent Orange exposure and 2,3,7,8-Tetrachlorodibenzo-p-Dioxin (TCDD) in human milk. Birth Defects Res. B Dev. Reprod. Toxicol. 104 (3), 129–139. 10.1002/bdrb.21145 26195119

[B60] Singh N. P. Nagarkatti M. Nagarkatti P. (2020). From suppressor T cells to regulatory T cells: how the journey that began with the discovery of the toxic effects of TCDD led to better understanding of the role of AhR in immunoregulation. Int. J. Mol. Sci. 21 (21), 7849. 10.3390/ijms21217849 33105907 PMC7660163

[B61] Singh N. P. Nagarkatti M. Nagarkatti P. S. (2007). Role of dioxin response element and nuclear factor-kappaB motifs in 2,3,7,8-tetrachlorodibenzo-p-dioxin-mediated regulation of Fas and Fas ligand expression. Mol. Pharmacol. 71 (1), 145–157. 10.1124/mol.106.028365 16940415

[B62] Singh N. P. Singh U. P. Guan H. Nagarkatti P. Nagarkatti M. (2012). Prenatal exposure to TCDD triggers significant modulation of microRNA expression profile in the thymus that affects consequent gene expression. PLoS One 7 (9), e45054. 10.1371/journal.pone.0045054 23024791 PMC3443208

[B63] Tischkau S. A. Jaeger C. D. Krager S. L. (2011). Circadian clock disruption in the mouse ovary in response to 2,3,7,8-tetrachlorodibenzo-p-dioxin. Toxicol. Lett. 201 (2), 116–122. 10.1016/j.toxlet.2010.12.013 21182907 PMC3039055

[B64] Viluksela M. Pohjanvirta R. (2019). Multigenerational and transgenerational effects of dioxins. Int. J. Mol. Sci. 20 (12), ijms20122947. 10.3390/ijms20122947 PMC6627869 31212893

[B65] Valdez K. E. Shi Z. Ting A. Y. Petroff B. K. (2009). Effect of chronic exposure to the aryl hydrocarbon receptor agonist 2,3,7,8-tetrachlorodibenzo-p-dioxin in female rats on ovarian gene expression. Reprod. Toxicol. 28 (1), 32–37. 10.1016/j.reprotox.2009.03.004 19490992 PMC2691863

[B66] Vázquez-Gómez G. Karasová M. Tylichová Z. Kabátková M. Hampl A. Matthews J. (2022). Aryl hydrocarbon receptor (AhR) limits the inflammatory responses in human lung adenocarcinoma A549 cells via interference with NF-κB signaling. Cells 11 (4), 707. 10.3390/cells11040707 35203356 PMC8870046

[B67] Vergara S. P. Lizama C. Brouwer-Visser J. Moreno R. D. (2011). Expression of BCL-2 family genes in germ cells undergoing apoptosis during the first wave of spermatogenesis in the rat. Andrologia 43 (4), 242–247. 10.1111/j.1439-0272.2010.01058.x 21486404

[B68] Wang L. Xu C. (2015). Role of microRNAs in mammalian spermatogenesis and testicular germ cell tumors. Reproduction 149 (3), R127–R137. 10.1530/REP-14-0239 25352684

[B69] Weber E. M. Algers B. Hultgren J. Olsson I. A. (2013). Pup mortality in laboratory mice--infanticide or not? Acta Vet. Scand. 55 (1), 83. 10.1186/1751-0147-55-83 24256697 PMC4176978

[B70] Wright E. J. De Castro K. P. Joshi A. D. Elferink C. J. (2017). Canonical and non-canonical aryl hydrocarbon receptor signaling pathways. Curr. Opin. Toxicol. 2, 87–92. 10.1016/j.cotox.2017.01.001 32296737 PMC7158745

[B71] Yao Q. Chen Y. Zhou X. (2019). The roles of microRNAs in epigenetic regulation. Curr. Opin. Chem. Biol. 51, 11–17. 10.1016/j.cbpa.2019.01.024 30825741

[B72] Zhang L. Duan H. B. Yang Y. S. (2017). Knockdown of Rap2B inhibits the proliferation and invasion in hepatocellular carcinoma cells. Oncol. Res. 25 (1), 19–27. 10.3727/096504016X14685034103914 28081729 PMC7840814

[B73] Zhang M. Buekens A. Jiang X. Li X. (2015). Dioxins and polyvinylchloride in combustion and fires. Waste Manag. Res. 33 (7), 630–643. 10.1177/0734242X15590651 26185164

[B74] Zhang T. Zhou X. Ren X. Zhang X. Wu J. Wang S. (2021). Animal Toxicology studies on the male reproductive effects of 2,3,7,8-Tetrachlorodibenzo-p-Dioxin: data analysis and health effects evaluation. Front. Endocrinol. (Lausanne) 12, 696106. 10.3389/fendo.2021.696106 34803904 PMC8595279

